# Overexpression of a *SOC1*-Related Gene Promotes Bud Break in Ecodormant Poplars

**DOI:** 10.3389/fpls.2021.670497

**Published:** 2021-05-25

**Authors:** Daniela Gómez-Soto, José M. Ramos-Sánchez, Daniel Alique, Daniel Conde, Paolo M. Triozzi, Mariano Perales, Isabel Allona

**Affiliations:** ^1^Centro de Biotecnología y Genómica de Plantas, Instituto de Investigación y Tecnología Agraria y Alimentaria, Universidad Politécnica de Madrid, Madrid, Spain; ^2^Departamento de Biotecnología-Biología Vegetal, Escuela Técnica Superior de Ingeniería Agronómica, Alimentaria y de Biosistemas, Universidad Politécnica de Madrid, Madrid, Spain

**Keywords:** poplar, MADS-box family transcription factors, dormancy, gibberellins, growth reactivation, SOC1, ecodormancy, bud break

## Abstract

Perennial species in the boreal and temperate regions are subject to extreme annual variations in light and temperature. They precisely adapt to seasonal changes by synchronizing cycles of growth and dormancy with external cues. Annual dormancy–growth transitions and flowering involve factors that integrate environmental and endogenous signals. MADS-box transcription factors have been extensively described in the regulation of *Arabidopsis* flowering. However, their participation in annual dormancy–growth transitions in trees is minimal. In this study, we investigate the function of *MADS12*, a *Populus tremula* × *alba SUPPRESSOR OF CONSTANS OVEREXPRESSION 1* (*SOC1*)-related gene. Our gene expression analysis reveals that *MADS12* displays lower mRNA levels during the winter than during early spring and mid-spring. Moreover, *MADS12* activation depends on the fulfillment of the chilling requirement. Hybrid poplars overexpressing *MADS12* show no differences in growth cessation and bud set, while ecodormant plants display an early bud break, indicating that *MADS12* overexpression promotes bud growth reactivation. Comparative expression analysis of available bud break-promoting genes reveals that *MADS12* overexpression downregulates the *GIBBERELLINS 2 OXIDASE 4* (*GA2ox4*), a gene involved in gibberellin catabolism. Moreover, the mid-winter to mid-spring RNAseq profiling indicates that *MADS12* and *GA2ox4* show antagonistic expression during bud dormancy release. Our results support *MADS12* participation in the reactivation of shoot meristem growth during ecodormancy and link *MADS12* activation and *GA2ox4* downregulation within the temporal events that lead to poplar bud break.

## Introduction

Woody perennial plants have acquired multiple adaptive mechanisms to coordinate their vegetative and reproductive growth with the seasonal weather changes (Cooke et al., 2012; Brunner et al., 2014). In temperate latitudes, trees switch between growth and dormancy at their shoot apical meristems to survive dehydration and freezing stress during winter months (Yordanov et al., 2014). Deciduous woody plants cease meristem activity to establish a dormant state before winter; this is called endodormancy. The meristem is rendered insensitive to growth-promoting signals until the chilling requirement fulfillment (Rohde et al., 2007). The inability to initiate growth clearly distinguishes between endodormancy and the subsequent stage, called ecodormancy, when tree buds recover growth capacity in late winter without showing changes in morphology, maintaining plants cold protected (Groover and Cronk, 2017; Maurya and Bhalerao, 2017). During ecodormancy, the tree has the capacity to resume growth and bud break. Cell elongation of preformed leaves inside the buds precedes new cell divisions (Rohde et al., 2007). Photoperiod and temperature are the main regulatory signals of *Populus* dormancy establishment and release (Wareing, 1956; Weiser, 1970; Ding and Nilsson, 2016; Singh et al., 2017). Changes in photoperiod are constant every year in a given location, while the temperature is more variable among the years. This variation in the temperature has been exacerbated by global warming, causing significant ecological and economic detriment. Warmer springs substantially advance leaf unfolding and flowering time in perennials. On the contrary, warmer winters compromise the chilling fulfillment, which results in a delay of bud break (Wang H. et al., 2020). A better understanding of the spring phenology's molecular control in perennials is crucial to avoid global warming damages and help perennials resist future climate.

The photoperiodic pathway drives *Arabidopsi*s flowering and poplar shoot growth (Shim et al., 2017; Singh et al., 2017; Triozzi et al., 2018). The CONSTANS/FLOWERING LOCUS T (CO/FT) module conserves the day length measurement function of *Arabidopsis* and poplar (Yanovsky and Kay, 2002; Bohlenius, 2006). Shoot growth resumption after winter correlates with activation of *FT1* and gibberellin (GA) biosynthetic genes (Rinne et al., 2011; Pin and Nilsson, 2012). More specifically, they encode members of the *GA3* and *GA20 oxidases*. GAs correlate with the activation of glucanase *GH17s* gene to reopen the plasmodesmata channels. They reinitiate the symplastic growth-promoting cell-to-cell signaling within the SAM (Rinne et al., 2011; Tylewicz et al., 2018). In poplar, GAs work parallel to the FT pathway controlling shoot elongation since high levels of bioactive GA during short-day (SD) conditions were sufficient to sustain shoot elongation growth (Eriksson et al., 2015; Singh et al., 2018). Furthermore, inactivation of GAs in *GA 2-OXIDASES* (*GA2ox*) overexpressing (OE) poplars produced extreme tree dwarfism (Zawaski et al., 2011). Additionally, the timing of bud break in poplar requires APETALA2/ethylene-like responsive factor, *Early Bud-Break 1* (*EBB1*), which is a positive regulator of bud break (Yordanov et al., 2014). *EBB1* overexpression shows early bud break, and downregulation delayed bud break relative to wild-type (WT) plants (Yordanov et al., 2014). Finally, it is essential to highlight those dynamics in genomic DNA methylation level involved in regulating the dormancy–growth cycle in trees (Santamaría et al., 2009; Conde et al., 2013, 2017a,b). A progressive reduction of genomic DNA methylation in the apex precedes growth in the apical shoot during the bud break. The induction in the apex of a chilling-dependent poplar *DEMETER-LIKE 10* (*DML10*) DNA demethylase causes a global reduction of DNA methylation levels before bud break (Conde et al., 2017a).

Multiple developmental pathways and stress responses in plants include MADS-box transcription factors (TFs), recently reviewed in Castelán-Muñoz et al. (2019). In trees, MADS-box has emerged as a crucial regulator of dormancy–growth transition during vegetative and reproductive development (Rodriguez-A et al., 1994; Hoenicka et al., 2008; Horvath et al., 2010; Leida et al., 2010; Kayal et al., 2011; Klocko et al., 2018; Singh et al., 2018; Falavigna et al., 2019; Wang J. et al., 2020). Genomic study of *Prunus persica evergrowing* (*eve*) mutant revealed a deletion of six *DORMANCY ASSOCIATED MADS-box* (*DAM*) genes essential for endodormancy maintenance (Rodriguez-A et al., 1994). Consistent with this role of DAM, overexpression of *Malus domestica MdDAMb* gene showed delayed bud break (Wu et al., 2017). Endodormancy establishment and maintenance in *Populus* and *M. domestica* require a gene phylogenetically related to *Arabidopsis SHORT VEGETATIVE PHASE* (*SVP*) (Wu et al., 2017; Singh et al., 2018). Opposite to DAM and SVP, heterologous overexpression in poplar of a silver birch *FRUITFUL* homolog, *BpMADS4*, prevented bud dormancy (Hoenicka et al., 2008). Seasonal expression studies identified MADS-box homologs *Arabidopsis SUPPRESSOR OF OVEREXPRESSION OF CO1* (*SOC1*) during winter dormancy (Voogd et al., 2015; Kitamura et al., 2016; Wang J. et al., 2020). *Arabidopsis SOC1* integrates vernalization, photoperiodic, aging, GAs, and flowering signals and promotes meristematic activity-initiated floral meristems (Moon et al., 2003; Andrés and Coupland, 2012; Immink et al., 2012). *SOC1-like* homologs display a seasonal expression pattern and form a heterocomplex with SVP and DAM homologs of *Arabidopsis*, kiwifruit trees, Japanese apricot, and sweet cherry (de Folter et al., 2005; Voogd et al., 2015; Kitamura et al., 2016; Wang J. et al., 2020). Functional analysis of tree *SOC1* homologs during growth dormancy transitions is minimal. Nevertheless, Voogd et al. (2015) studied the phenology of kiwifruit *AcSOC1i*-, *AcSOC1e*-, and *AcSOC1f* OE lines, finding that only *AcSOC1i* presented earlier bud break than control plants. Even though SOC1, SVP, and DAM homologs could coexpress and interact, some members might have unique function during dormancy.

In this study, we investigate the role of the poplar *MADS12* gene in the dormancy–growth transition. Our phylogenetic analysis and protein sequence comparison indicate that MADS12 belongs to the SOC1 clade. Our gene expression analysis demonstrates *MADS12* induction once chilling is fulfilled, showing a peak of expression before bud break. Moreover, we study the performance of *MADS12* OE lines (OE3 and OE5) and WT plants, under phenological assays. Our findings show that hybrid poplar OE *MADS12* downregulates *GA2ox4* gene and promotes early bud break during ecodormancy.

## Materials and Methods

### Plant Material and Growth Conditions

The hybrid poplar *Populus tremula* × *alba* INRA clone 717 1B4 was used as the experimental model. Poplar plantlets were grown *in vitro* in Murashige and Skoog (MS) medium 1B (pH 5.7) supplemented with 2% sucrose and with indole acetic and indole butyric acids (0.5 mg/L) containing 0.7% (w/v) plant agar under long-day (LD) 16-h light/8-h dark and 22°C conditions.

For the phenological assays, *in vitro*-cultivated poplars of WT and four selected independent *MADS12* OE lines, OE1, OE3, OE5, and OE7, were transferred to pots containing blond peat, pH 4.5, and grown under LD and 22°C conditions for 3 weeks. The plants were fertilized once every 2 weeks with a solution of 1 g/L of Peters Professional 20-20-20 in LD conditions and 20-10-20 in SD conditions (Comercial Química Massó, Barcelona, Spain). Growth cessation and bud set were induced by exposing plants to SD conditions at 22°C (8-h light/16-h dark) during 8 or 10 weeks. Bud set progression was graded by scoring from stage 3 (fully growing apex) to stage 0 (fully formed apical bud) according to Rohde et al. (2010). Winter conditions were emulated by treating plant under SD 4°C for 4 or 6 weeks to fulfill the chilling requirement. Finally, plants were transferred back to LD 22°C to monitor bud break. The regrowth was scored according to the six developmental stages of bud break (stages 0 to 5) according to Johansson et al. (2014). Phenological assays to evaluate bud break of ecodormant plants were performed twice. In the first round, we assayed *MADS12* OE lines OE1, OE5, and OE7 lines and WT. We observed that the highest level of *MADS12* expression correlates with the earliest bud break. In the second round, we assayed only the *MADS12* OE3 and OE5 lines showing the highest *MADS12* expression levels and WT to increase the number of plantlets to make two biological replicates.

### Generation of T1 *MADS12* Overexpressing Lines

The *MADS12* coding region (CDS) was amplified from hybrid poplar using gene-specific primers with attB sites (Supplementary Table 1). For polymerase chain reaction (PCR), Phusion DNA Polymerase (Thermo Fisher Scientific, Massachusetts, USA) was used, and the PCR products were purified and inserted into pDONR207 (Life Technologies, Carlsbad, CA, USA). Insertions in the resulting entry clones were sequence verified. The Gateway cassettes carrying *MADS12* CDS were then transferred into the destination binary vectors pGWB15 (Nakagawa et al., 2007). These constructs were transferred into *Agrobacterium tumefaciens* strain GV3101/pMP90 (Koncz and Schell, 1986). Hybrid poplar was transformed via an *Agrobacterium*-mediated protocol described previously by Gallardo et al. (1999) with few modifications. Briefly, poplar leaves and stem explants were cultured in minimum inhibitory concentration (MIC) medium, MS medium 1B supplemented with 0.01 mg/L of thidiazuron (TDZ) and 1 mg/L of 2,4-dichlorophenoxyacetic acid in the dark for 48 h. Then explants were placed in an agrobacterium suspension for 15 min and blotted onto sterile filter paper to remove excess bacteria. After 2 days, explants were transferred to MISCT medium composed of MS 1B supplemented with 0.02 mg/L of TDZ, 1 mg/L of 2,4-dichlorophenoxyacetic acid, 250 mg/L of cefotaxime, and 50 mg/mL of kanamycin for 4 weeks in darkness. This medium was used for decontamination and induction of callus growth. After size reached about 0.5 mm^2^, calli were moved to MIBS medium supplemented with 0.05 mg/mL of alpha-naphthalene acetic acid (NAA), 0.004 mg/L of TDZ, 250 mg/L of cefotaxime, and 50 mg/L of kanamycin to select transformant calli and induce shoot formation. Once shoots grow, they are transferred to MEMS, MS 1B supplemented with 125 mg/L of cefotaxime, kanamycin (50 mg/L), and 0.5 mg/L of indole-3-acetic acid (IAA) to induce root formation. Regenerated plantlets were propagated in MEMS medium and maintained *in vitro* growth conditions (LD 22°C). *MADS12* expression level of individual T1 OE lines was analyzed by reverse transcription-PCR (qRT-PCR).

### Plant Material for Gene Expression Studies

To investigate if *MADS12* activation is dependent on the fulfillment of the chilling requirements, gene expression analysis was performed on RNA obtained from cuttings collected from five 6-year-old hybrid poplars (*P. tremula* × *alba*) growing under natural conditions as reported earlier by Conde et al. (2017a).

Annual expression patterns for poplar *MADS12* were initially determined in 2-year-old poplar branches (*Populus alba*) from adult trees growing under natural conditions in Madrid (Spain) (Conde et al., 2017b).

For comparative gene expression analysis among hybrid poplar *MADS12* OE lines, OE3 and OE5, and WT, RNA was obtained from a pool of six ecodormant apical buds grown for 5 days under LD at 22°C, for each biological sample.

Mid-winter to mid-spring transcriptional profiles, used for making heat maps, were collected from hybrid poplar apical bud samples grown in natural conditions in Madrid (Spain). Normalized expression data are available in the gene atlas from Phytozome https://phytozome.jgi.doe.gov/pz/portal.html (Conde et al., 2019).

### RNA Extraction, cDNA Synthesis, and qRT-PCR Analysis

RNA extraction was performed adding to frozen bud cetyltrimethylammonium bromide (CTAB) extraction buffer with 2% β-mercaptoethanol at 65°C for 5–8 min, following by three washes with chloroform. After that, 7.5 M LiCl_2_:50 mM EDTA was added, and samples were precipitated overnight at 4°C. The next day, RNA was collected by centrifuging at 10,000 RPM for 20 min, the supernatant was discarded, and the pellet was resuspended using NR buffer, and RNA was purified according to the manufacturer's instructions of NZY RNA purification kit (NZYTech, Lisboa, Portugal). A total of 500 ng of RNA was retrotranscribed to cDNA using the Maxima First Strand cDNA Synthesis kit from Thermo Scientific (Thermo Fisher Scientific, Massachusetts, USA), and qRT-PCR was performed as described earlier (Ramos-Sánchez et al., 2019). The gene-specific primers used in these studied are listed in Supplementary Table 1.

### Phylogenetic Analysis and Sequence Alignment

*Arabidopsis* protein sequences were obtained from TAIR10 website (www.arabidopsis.org), and *Populus trichocarpa* protein sequences from (Phytozome v10.3, www.phytozome.net). To construct the phylogenetic tree, protein sequences were aligned with MAFFT E-INS-I algorithm; ProtTest 2.4 was used to select the best evolutionary model (JTT+I+G+F; 5 categories rate; alpha = 1.213); the phylogeny was inferred with RAxML algorithm (perform bootstrap 1000). Tree representation was made using iTol (https://itol.embl.de/) and selecting a bootstrap < 600, and clade coloring was performed according to phylogenetic relationships with *Arabidopsis MADS-box* genes. Multiple sequence alignment was inferred using ClustalW. MADS-box protein domains were annotated as described in Trainin et al. (2013).

### *In Silico* Analysis of *GA2ox4* Promoter

Promoter sequence (2 kb) of *GA2ox4* was obtained from ASPENDB (http://aspendb.uga.edu/) and analyzed using the Plant promoter analysis navigator 3.0 (http://plantpan.itps.ncku.edu.tw/). MADS-box TF binding sides were selected and examined to search putative TFs that were identified using phytozome database (https://phytozome.jgi.doe.gov/pz/portal.html).

### *MADS12* Coexpression Analysis

Genes coexpressed with *MADS12* were identified using the Pearson Algorithm on bud mid-winter to mid-spring RNAseq dataset applying correlation coefficient higher than 0.9. We identified 258 genes in apical bud dataset coexpressed with *MADS12*. Gene expression values for each gene were normalized being the maximum of gene expression 1. From a total of 258 *MADS12* coexpressed genes, a total of 242 unique homologs to *Arabidopsis* genome were identified.

## Results

### Poplar SOC1 Clade Displays Seven Members

**Figure 1:**
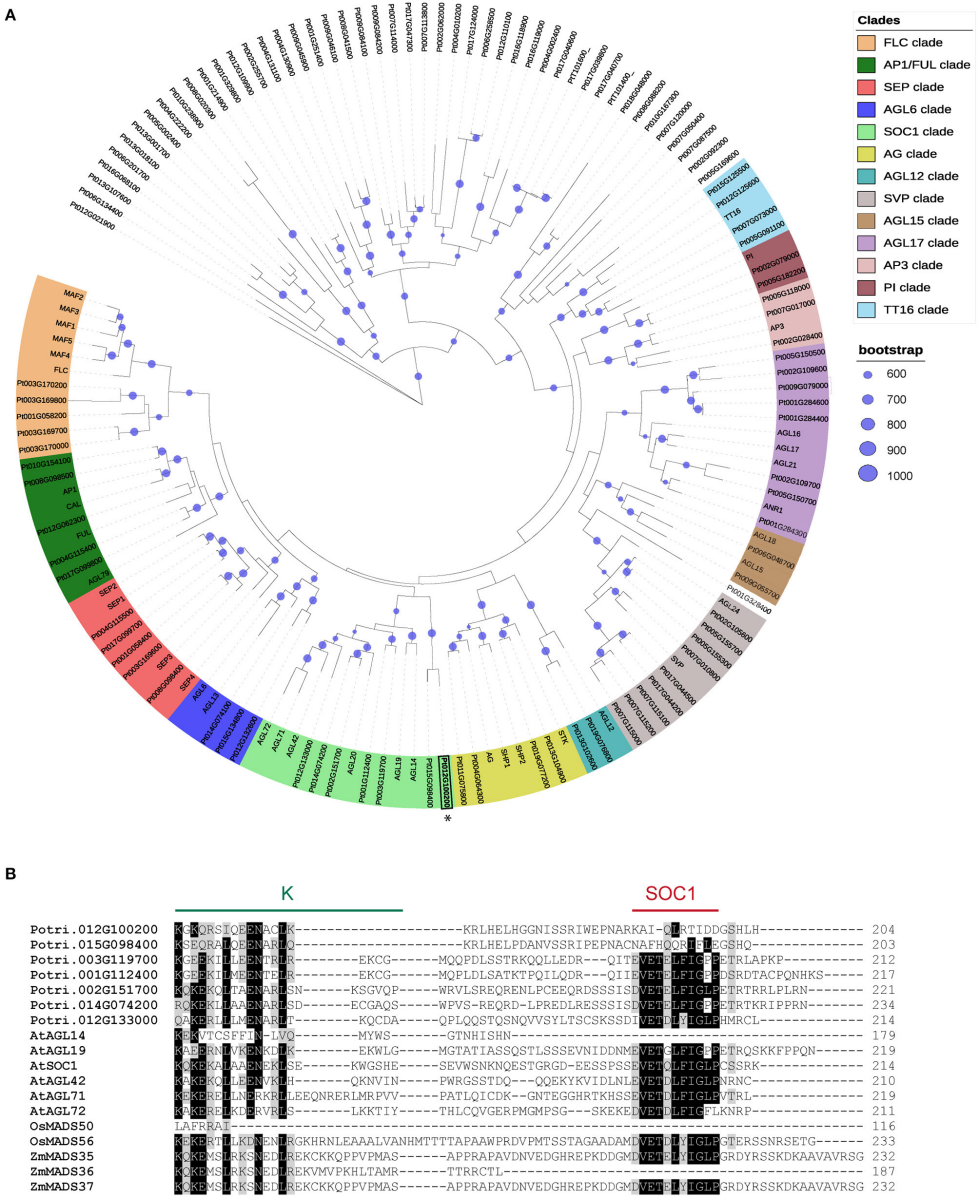
Identification of poplar SOC1-like proteins. **(A)** Phylogenetic tree analyses of MADS-box proteins from *Arabidopsis* and poplar. Maximum likelihood tree obtained using the RAxML tool with 1,000 bootstrap replicates. The right panel indicates the color code of the clades and bootstrap values. Clades are named FLOWERING LOCUS C (FLC), APETALA1/FRUITFUL (AP1/FUL), SEPALLATA (SEP), AGAMOUS-LIKE 6 (AGL6), SOC1, AG, AGL12, SVP, AGL15, AGL17, AP3, PISTILLATA (PI), and TRANSPARENT TESTA16 (TT16). **(B)** MAFF alignment of *Populus, Arabidopsis*, rice, and maize SOC1-like proteins. The alignment shows part of the K domain (green ink) and the SOC1 motif (red ink). Black boxes indicate identical amino acids. Gray boxes indicate conservative amino acid substitutions.

MADS-box TF gene family displays a large number of uncharacterized members in poplar. We investigated the phylogenetic relationship of *MADS-box* genes in *Arabidopsis thaliana* and *Populus trichocarpa* by generating a maximum likelihood phylogenic tree using all annotated poplar and *Arabidopsis* full-length MADS-box protein sequences. Thirteen major lineages within MIKC-types MADS-box have been resolved and named according to the *Arabidopsis* gene terminology: FLC/MAF, AP1/FUL, SEP, AGL6, SOC1, AG, AGL12, SVP, AGL15, AGL17, AP3, PI, and TT16. Also, we found two clusters that belong to MIKC-type proteins without any *Arabidopsis* orthologous (Figure 1A). Even though several *SOC1-like* genes were associated with dormancy release in trees (Voogd et al., 2015; Kitamura et al., 2016; Wang J. et al., 2020), the poplar SOC1-like members remain uncharacterized during dormancy phenology. We performed protein comparison of identified SOC1-like members of *Arabidopsis*, poplar, rice, and maize, revealing a high sequence similarity among MADS-box (M) domain and less conserved intervening (I) and keratin-like (K) domains (Supplementary Figure 1). Interestingly, most SOC1-like proteins display conserved C-terminal motif; however, there are members that lack this C-terminal sequence in monocots and dicots (Figure 1B). Together, these analyses indicate that SOC1 members might have originated from a common MIKC-type MADS-box ancestor; nevertheless, they have evolved to a novel C-terminal sequence. Since all *Arabidopsis SOC1-like* genes promote flowering transition, the SOC1 C-terminal motif is not critical for flowering (Schonrock, 2006; Lee and Lee, 2010; Dorca-Fornell et al., 2011; Pérez-Ruiz et al., 2015).

### *MADS12* Activation Occurs Once the Chilling Requirement Has Been Fulfilled

**Figure 2:**
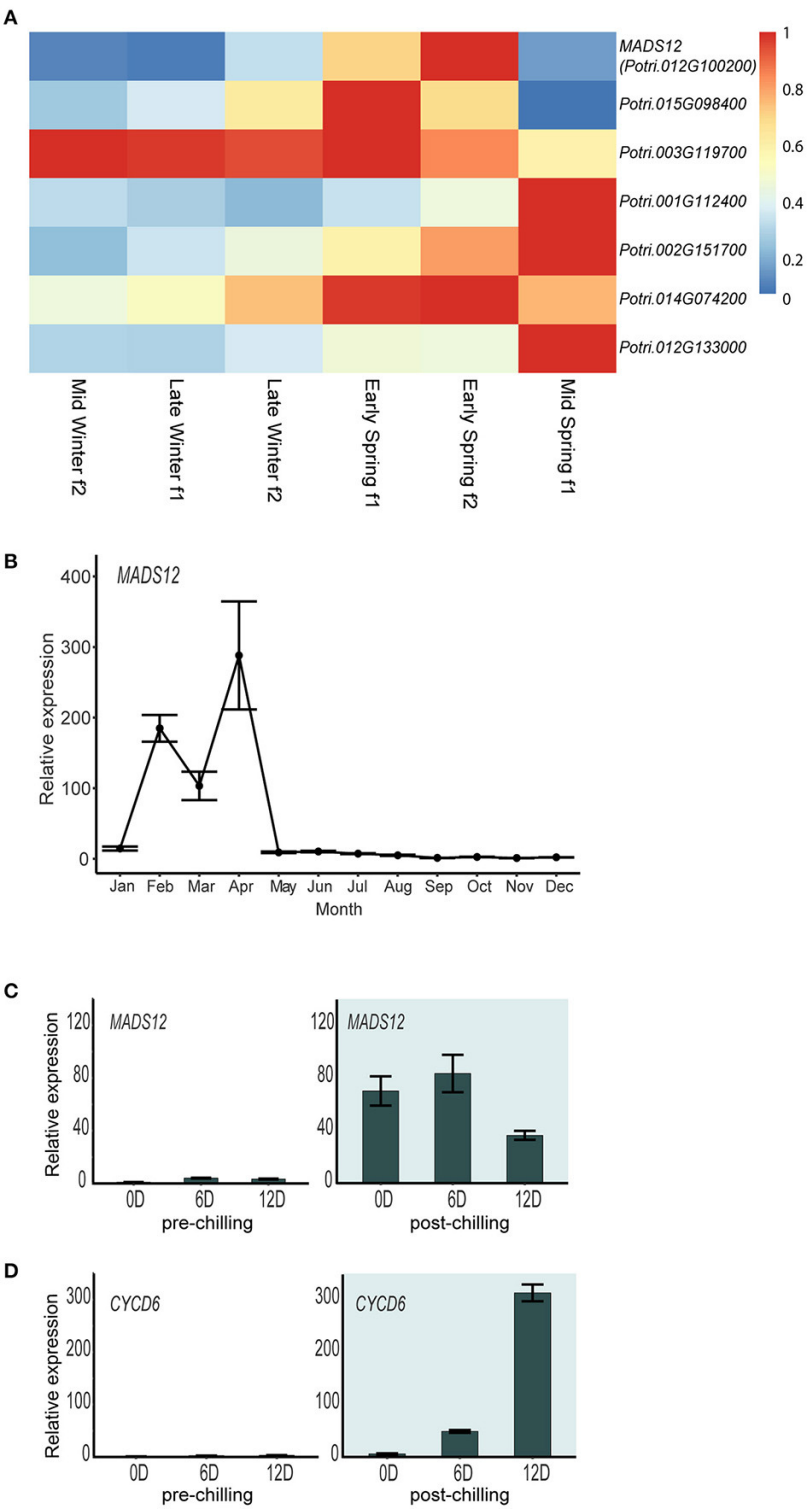
Chilling requirement fulfillment is a prerequisite for *MADS12* activation. **(A)** Heatmap showing normalized expression of hybrid poplar SOC1-like genes from mid-winter to mid-spring in apical buds. Groups are named “early,” “mid,” or “late” when sampling dates were within the first, second, or third month of each season, respectively, following the Northern Meteorological Seasons dates. “f1” or “f2” mean fortnight-1 and fortnight-2 within the month (Conde et al., 2019). **(B)** *MADS12* annual expression pattern measured by qRT-PCR in 2-year-old poplar branches over 1 year. *Ubiquitin7* is used as the housekeeping gene. Plotted values and error bars are fold-change means ± s.d. of two biological replicates. Note: *MADS12* shows the highest mRNA accumulation during mid-winter–spring period. **(C,D)** qRT-PCR analysis of *MADS12* **(C)** and *CYCD6* **(D)** genes in dormant shoot apex of pre-chilling and post-chilling cuttings grown under LD and 22°C conditions. *Ubiquitin7* is used as the housekeeping gene. Plotted values and error bars are fold-change means ± s.d. of two biological replicates. The x-axis scale is indicated in days. Pre-chilling and post-chilling data are shown in white and green backgrounds, respectively.

To understand the expression pattern of poplar *SOC1-like* genes during dormancy to growth shift, we studied their temporal expression in the Phytozome public repository, which deposited RNAseq-based gene expression analyses of hybrid poplar apical buds grown under natural conditions (Conde et al., 2019). All these genes display a particular mid-winter to mid-spring pattern of gene expression in shoot apical buds, showing a temporal specialization (Figure 2A). We focused on *Potri.012G100200* gene, called *MADS12*, which shows a low expression level during the winter followed by a transitory expression peak with its maximum at Early Spring f2 (Figure 2A). *MADS12* is a spring gene activated during the shoot growth resumption period (Figure 2B). The repression of *MADS12* observed during the winter suggested that its activation depends on fulfilling the chilling requirement (Figure 2B). We investigated *MADS12* expression in dormant buds of pre-chilling and post-chilling cuttings, after moving them to growth-promoting conditions during 0, 6, and 12 days. Our qRT-PCR analysis confirmed that *MADS12* is induced once the chilling requirement has been fulfilled (Figure 2C). Moreover, *MADS12* shows its maximal expression at 6 days (83-fold) and decreasing ~50% at 12 days (Figure 2C). However, the expression of *CYCLIN C 6* (*CYC6*) gene, used as marker of cell proliferation (Conde et al., 2017a), peaks at 12 days (Figure 2D), suggesting that *MADS12* induction precedes the cell proliferative stage. These results suggest that *MADS12* could be involved in the resumption of growth after chilling period.

### *MADS12* Overexpression Promotes Shoot Growth Reactivation of Ecodormant Plants

To assess the functional role of *MADS12* during dormancy–growth cycles, we generated 10 independent OE hybrid poplar lines, characterizing their *MADS12* expression levels by qRT-PCR (Supplementary Figure 2). Because of the highest *MADS12* expression level, we selected OE3 and OE5 lines to examine the growth and apical bud phenology in growth chambers under photoperiodic and temperature conditions that mimic seasonal transitions autumn–winter–spring (Rohde et al., 2010). Phenology of WT and *MADS12* OE3 and OE5 genotypes did not show any differences in growth cessation and bud set, indicating that *MADS12* overexpression does not impair these transitions (Supplementary Figures 3A,B).

**Figure 3:**
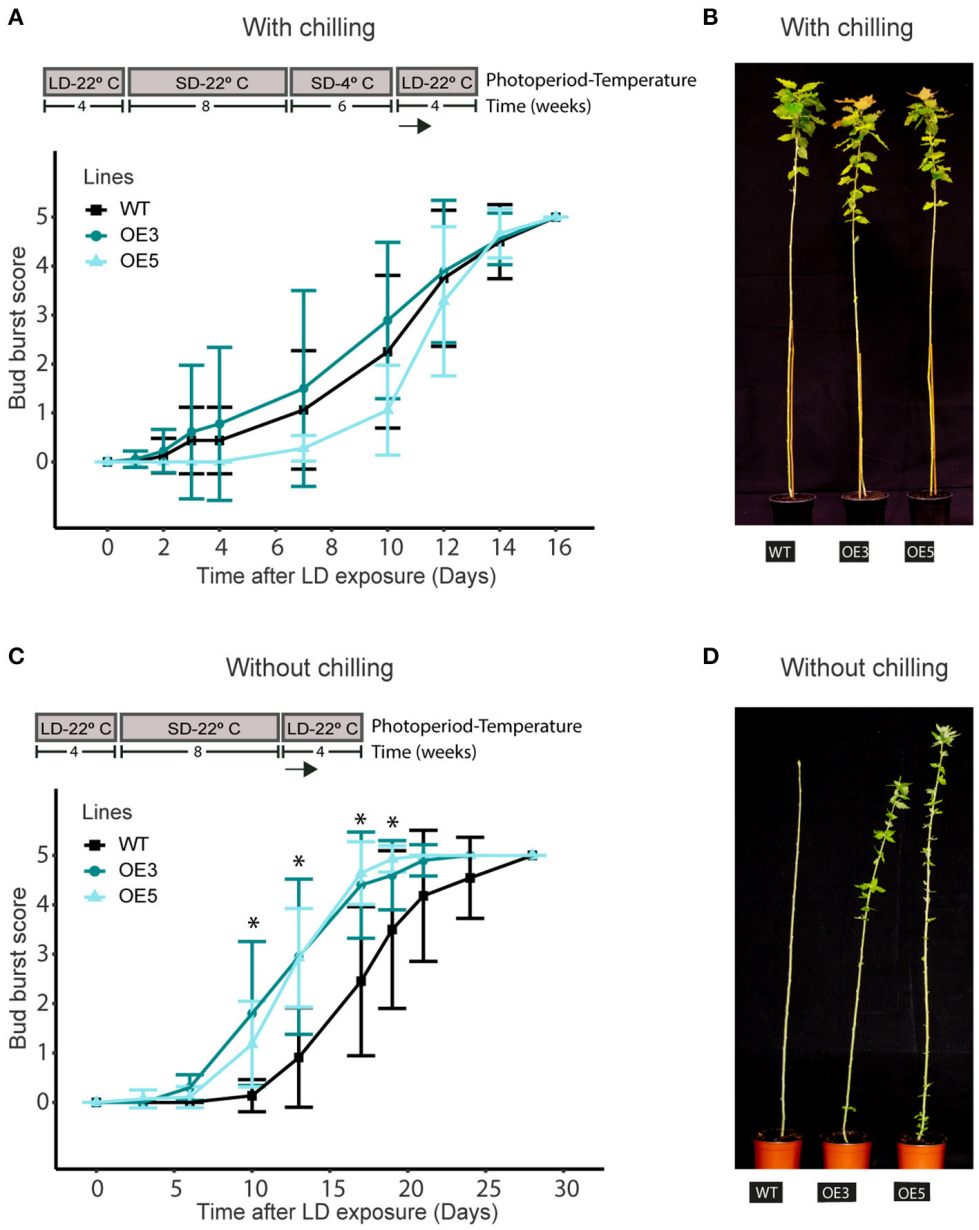
*MADS12* overexpression promotes early bud break of ecodormant poplars. Bud break scoring of hybrid poplar *MADS12* overexpressing OE3 and OE5 lines and wild type after transferring to LD and 22°C conditions **(A,B)** with chilling treatment and **(C,D)** without chilling treatment. **(B,D)** Representative picture of bud break differences in **(B)** plants after 15 days of LD exposure and **(D)** plants after 10 days of LD exposure. Values represent the mean of the bud score measure of *n* = 10–15 plants. Significant differences between overexpressing (OE) and wild type (WT) were analyzed using Tukey test, *p < 0.05. Top panels indicate photoperiodic and temperature conditions used.

To evaluate if *MADS12* OE plants show changes in endodormancy release, we subjected those plants to 8 weeks of SDs at 22°C followed by 4 or 6 weeks of SD at 4°C. Subsequently, we shifted them to LDs at 22°C for monitoring their bud break (Conde et al., 2017a). Noticeably, all genotypes tested remained dormant under growth-promoting conditions after 4 weeks of SD at 4°C, although they resumed growth after 6 weeks of SD at 4°C (Supplementary Figures 3A,C). We observed no differences in bud break scores among genotypes, suggesting that chilling requirement fulfillment caused an equal reactivation of shoot growth (Figures 3A,B). Once trees entered in endodormancy, WT and OE plants have same chilling requirement, and *MADS12* overexpression does not induce endodormancy release.

Afterward, we tested whether *MADS12* OE could restore growth on plants that had already ceased growing and set buds without any chilling treatment. We arranged a new set of WT, *MADS12* OE3, and OE5 plants in LD at 22°C for 4 weeks and placed them under SD at 22°C for 8 or 10 weeks. Then, plants were subjected again to LD at 22°C, and bud break was monitored (Figure 3C and Supplementary Figure 3D). After 10 weeks in SD at 22°C, WT and most *MADS12* OE3 and OE5 plants entered endodormancy; therefore, they could not resume growth under favorable environmental conditions without chilling (Supplementary Figure 3D). This observation indicates that *MADS12* overexpression does not restrict the plants from reaching the endodormant state and that they could not break endodormancy without chilling. Finally, plants subjected to SD at 22°C conditions for 8 weeks ceased growth and set buds; however, they could not reach the endodormant state since plants resumed total shoot growth under LD at 22°C without chilling (Figure 3C). Significantly, the apical shoot of *MADS12* OE3 and OE5 lines restored full growth ~10 days earlier than WT plants (Figures 3C,D). Furthermore, a phenological assay of additional lines confirmed that the lower the *MADS12* overexpression level, the lower the differences in bud break observed (Supplementary Figure 4). Collectively, these results indicate that *MADS12* overexpression promotes early bud break of ecodormant plants under favorable environmental conditions.

### *GA2ox4* Is Downregulated in Ecodormant *MADS12* Overexpressing Plants

**Figure 4:**
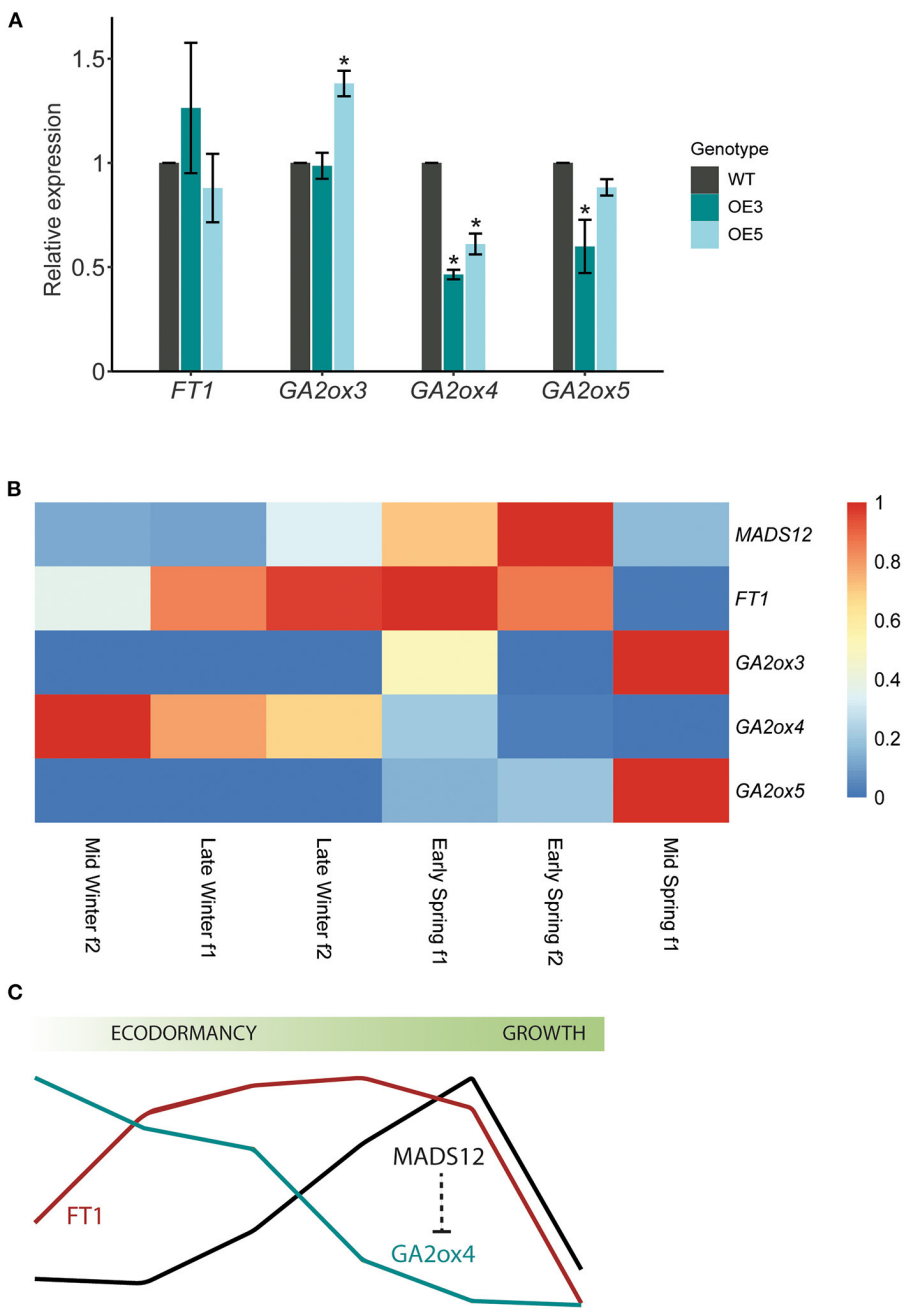
*GA2ox4* is downregulated in *MADS12* overexpressing lines during bud dormancy release. **(A)** qRT-PCR analysis of *FT1, GA2ox3, GA2ox4*, and *GA2ox5* genes in ecodormant *MADS12* overexpressing OE3 and OE5 and wild-type (WT) apices collected after 5 days in long day (LD) and 22°C treatment. *Ubiquitin7* is used as the housekeeping gene. Plotted values and error bars are fold-change means ± s.d. of two biological replicates. Asterisks (*) represent statistical differences assessed by one-way ANOVA followed by Tukey *post-hoc* test (*p* < 0.05). **(B)** Heatmap showing RNAseq expression data of *MADS12, FT1, GA2ox3, GA2x4*, and *GA2ox5* genes in apical buds from mid-winter f2 to mid-spring f1. **(C)** Schematic representation of *MADS12* and *GA2ox4* antagonist expression pattern during bud dormancy release.

Our phenological assays revealed an accelerated shoot growth resumption of *MADS12* OE lines with respect to WT, suggesting an improved growth-stimulating capacity. We hypothesize that MADS12 OE3 and OE5 might differentially express shoot growth-promoting genes with respect to WT. To test this, we collected apical buds of *MADS12* OE3, *MADS12* OE5, and WT plants treated with 8 weeks of SD at 22°C and exposed during 5 days to LD at 22°C conditions, before the observed phenotypic differences (Figure 3C). In poplar, one of the main factors involved in shoot growth is FT (Bohlenius, 2006; Hsu et al., 2011). qRT-PCR analysis did not show differences in *FT1* expression in *MADS12* OE3 and *MADS12* OE5 lines with respect to WT (Figure 4A). We did not detect *FT2* in these bud tissues (data not shown). This result indicates that an FT-independent pathway controls the activation of shoot growth. Bioactive GAs act as an FT parallel pathway to control poplar shoot growth (Eriksson et al., 2015). *GA2ox* genes cause the inactivation of bioactive GAs (Rieu et al., 2008). Only *GA2ox3, GA2ox4*, and *GA2ox5* are expressed in the apical shoot, and RNAi downregulation of *GA2ox4* and *GA2ox5* promotes poplar aerial shoot growth (Gou et al., 2011). Our qRT-PCR analysis showed that *GA2ox4* and *GA2ox5* are downregulated in *MADS12* OE3 and OE5 with respect to WT, while only *GA2ox4* is found to be significantly repressed (Figure 4A). *GA2ox3* shows inconsistent differences in *MADS12* OE3 and OE5 with respect to WT (Figure 4A). These results point out that an increase in bioactive GAs could cause the accelerated growth resumption. Accordingly, the mid-winter to mid-spring RNAseq analysis showed an opposite temporal expression pattern, with *GA2ox4* and *GA2ox5* being greatly downregulated when *MADS12* is highly expressed (Figure 4B). This result supports the antagonistic role of *MADS12* over *GA2ox4* within the temporal events that lead to poplar bud break.

## Discussion

MADS-box homologs to *Arabidopsis* SOC1 have been identified in trees; however, their function remains very little understood. *Arabidopsis SOC1* is expressed in the shoot apical meristem and plays a key role during floral transition, integrating vernalization, photoperiod, GAs, and the aging pathways (Amasino, 2010; Lee and Lee, 2010). Furthermore, *Arabidopsis SOC1*-related genes, *AGL14, AGL19, AGL42, AGL71*, and *AGL72*, are also implicated in the control of the floral transition (Schonrock, 2006; Dorca-Fornell et al., 2011; Pérez-Ruiz et al., 2015). Our phylogenetic tree of poplar and *Arabidopsis* MADS-box proteins identifies a SOC1 clade including seven poplar SOC1-like family members (Figure 1A). These seven poplar *SOC1-like* genes show a specific expression pattern during mid-winter to mid-spring, suggesting their role in dormancy release (Figure 2A). Among them, MADS12 and Potri.015G098400 do not conserve a C-terminal SOC1 motif, suggesting that MADS12 C-terminal domain undergoes a divergent evolution (Figure 1B). The functional importance of C-terminal SOC1 motif is still unclear. Structure-function analysis of *Arabidopsis* with intragenic mutations in the *SOC1* gene shows that deletion of C-terminal and part of the K domain renders weak suppression of early flowering of FRIGIDA mutants (Lee et al., 2000, 2008). This observation and the fact that *AGL14*, also lacking the C-terminal SOC1 motif, is required for *Arabidopsis* flowering (Pérez-Ruiz et al., 2015) indicate that the C-terminal SOC1 motif is not essential for SOC1 and its homologs function in flowering.

Functional analyses of three *SOC1* homolog genes in the kiwifruit tree, which exhibited a C-terminal SOC1 motif, resulted in only *AcSOC1i* overexpression that promoted early bud break under ecodormant conditions (Voogd et al., 2015). *MADS12* OE lines also display an accelerated bud break of ecodormant poplars. Moreover, the seasonal expression pattern of *AcSOC1i* resembles one of the *MADS12* showing a seasonal induction at the end of the winter (Figures 2A,B; Voogd et al., 2015). These two studies point out that *AcSOC1i* and *MADS12* play identical functions during dormancy phenology, despite C-terminal SOC1 motif differences. Similar to our hybrid poplar *MADS12* OE lines, overexpression of *Arabidopsis SOC1* in poplar did not show phenotypical differences during vegetative shoot growth, but phenological assays were not performed (Bruegmann and Fladung, 2019). Additional functional studies of poplar *SOC1-like* genes are needed.

This work shows that chilling requirement fulfillment is necessary for *MADS12* activation, as it does for *FT1, EEB1*, and *DML10*, already described as bud break promoter genes in poplar (Hsu et al., 2011; Yordanov et al., 2014; Conde et al., 2017a). Our results show that *MADS12* participates in ecodormancy release, concurring with a single-nucleotide polymorphism's (SNP's) presence associated with bud flush (Evans et al., 2014). We find that ecodormant *MADS12* OE lines resume growth under LDs and warm temperatures 10 days earlier than WT, consistent with its possible function as a growth inductor during ecodormancy. Our phenological assays indicate that bud break of *MADS12 OE* poplars has no differences to WT after chilling fulfillment. Possibly, *MADS12* OE phenotype would have been masked by the induction of endogenous *MADS12* after chilling. To test this hypothesis, bud break analysis of *MADS12* knockout lines should be performed. Another possibility is that the levels of a transcriptional coregulator limit the activity of *MADS12* OE plants *in vivo*. MADS-box transcriptional regulation operates forming heterocomplexes (de Folter et al., 2005). We found that *AGAMOUS-LIKE MADS-BOX PROTEIN AGL16* (*AGL16-like*) TF coexpressed with *MADS12* (Supplementary Figures 5, 6A, and Supplementary Table 3). Moreover, *AGL16-like* expression is not induced in *MADS12* OE lines (Supplementary Figure 6B). Thus, we proposed that chilling dependent bud break in *MADS12* OE lines could be conditioned by a limiting factor such as *AGL16-like*. Future protein–protein interaction and coexpression assays in transgenic poplar will sort this out.

During the ecodormancy stage before bud break, reactivation of growth correlates with upregulation of GA biosynthetic genes and simultaneous downregulation of GA catabolic genes, particularly *GA2ox4* (Karlberg et al., 2010). Likewise, bud break correlates with downregulation of GA2 oxidases in *Vitis vinifera* and sweet cherry (Zheng et al., 2018; Vimont et al., 2019). Exogenous addition of GAs to winter buds promoted poplar bud break (Rinne et al., 2011). A functional study demonstrate that the oveexpression of a Gibberellin Oxidase gene caused delayed bud break pointing to GA signaling involvement during bud break (Singh et al., 2018). Recent work showed that *MADS12* activates *PIN5b* in *Populus deltoides* × *euramericana* stem tissue during full-growth conditions (Zheng et al., 2020). Unexpectedly, our qRT-PCR analysis showed that *PIN5b* expression is significantly repressed in both *MADS12* OE3 and OE5 than WT, suggesting an impaired polar auxin efflux in *MADS12* OE ecodormant buds (Supplementary Figure 7). The differences in seasonal stages or *Populus* ecotypes might explain this contrasting activity of *MADS12*. *Arabidopsis* PIN5 localizes in endoplasmic reticulum (ER) membrane and mediates polar auxin efflux from the cytosol to ER (Mravec et al., 2009). This intracellular auxin transport plays a role in regulating auxin homeostasis by compartmentalizing cellular auxin pools (Barbez and Kleine-Vehn, 2013). Thus, repression of *PIN5b* in ecodormant buds could increase cytoplasmatic auxin pool. That could contribute to the faster shoot growth resumption capacity observed in MADS12 OE plants, along with GAs. Phenological assays of *PIN5b* RNAi lines are necessary to investigate its role during winter-to-spring transition.

An increasing amount of evidence points out that dormancy and flowering transition sharing conserved regulatory elements (Maurya and Bhalerao, 2017; Triozzi et al., 2018; Falavigna et al., 2019). In *Arabidopsis* shoot apical meristem, SOC1 is the major hub in regulatory networks underlying flowering (Immink et al., 2012). SOC1 antagonizes SVP repressive action over *GA20 oxidases 2* (*GA20ox2*), promoting GA biosynthesis and flowering induction at the shoot apex (Andrés et al., 2014). It is unknown if this model could be conserved during dormancy to growth transition. An inspection of public mid-winter to mid-spring RNAseq data indicates that *SVL* shows an opposite expression pattern with *SOC1*-like genes encoded by *Potri.001G112400* and *Potri.012G13300*, and *GA20 oxidases 3, 5*, and *6*, but not with *MADS12* (Supplementary Figure 8). This observation suggests a possible interplay between *SOC1*-like genes and SVL to modulate GA biosynthesis in poplar during dormancy to growth transition. However, MADS12 might operate in an SVL-independent manner.

Our results revealed that ecodormant *MADS12* OE poplars show accelerated bud break and *GA2ox4* repression. Moreover, the RNAseq mid-winter to mid-spring profiling demonstrates that *MADS12* and *GA2ox4* show opposite expression patterns during dormancy release. *In silico* analysis of *GA2ox4* promoter identified 10 potential MADS-box-binding elements, supporting the idea that *GA2ox4* could be transcriptionally repressed by *MADS12* (Supplementary Table 2). Future transactivation assays should test this hypothesis. It has been shown that RNAi downregulation of *GA2ox4* increased shoot growth in poplar; however, it is unknown whether *GA2ox4* RNAi lines could reactivate earlier shoot growth of ecodormant plants (Gou et al., 2011; Singh et al., 2018). Phenological assays of *GA2ox4* RNAi lines are critical to sort the importance of downregulation of GA catabolism during winter-to-spring transition. We propose that the seasonal function of *MADS12* is to promote growth reactivation during ecodormancy by downregulating *GA2ox4* (Figure 4C). Activation of GA signaling promotes reactive oxygen species (ROS) signaling and bud break in Japanese apricot (Zhuang et al., 2013). A transitory activation of oxidative stress has been proposed to play a pivotal role in dormancy in several plant models (Beauvieux et al., 2018). Whether downregulation of *GA2ox4* by *MADS12* promotes GAs and ROS signaling should be explored in poplar.

## Data Availability Statement

The original contributions presented in the study are included in the article/Supplementary Materials, further inquiries can be directed to the Corresponding authors.

## Author Contributions

DG-S, JR-S, DA, DC, PT, and MP performed the experiments. All authors participated in the design of the experiments and in the discussions described here. DG-S, MP, and IA wrote the manuscript.

## Conflict of Interest

The authors declare that the research was conducted in the absence of any commercial or financial relationships that could be construed as a potential conflict of interest.
