# Supplementary material for: Overexpression of a SOC1-Related Gene Promotes Bud Break in Ecodormant Poplars
Source: Front Plant Sci. 2021 May 25;12:670497. doi: 10.3389/fpls.2021.670497 (PMC8185274; doi:10.3389/fpls.2021.670497)
Supplement: Supplementary file 1 [file Data_Sheet_1.PDF]

Figure S1. **MAFF alignment of *Populus*, *Arabidopsis*, rice, and maize SOC1 like proteins**. Black boxes indicate identical amino acids. Gray boxes indicate conservative amino acid substitutions. MADS box domains (M, blue ink), I domains (I, black ink) and a part of the K domains (K, green ink) are shown.

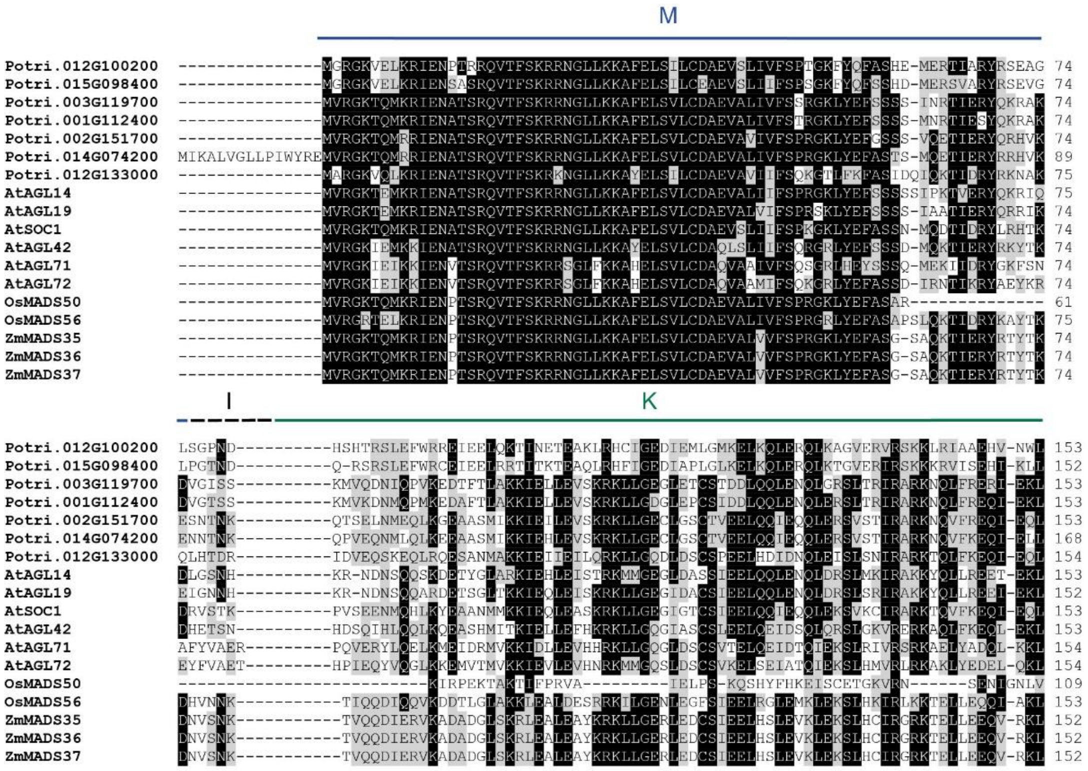

Figure S2. Characterization of *MADS12* transgenic lines. qRT-PCR analysis of *MADS12* overexpressing lines and wild-type plants. *Ubiquitin7* is used as the housekeeping gene. Plotted values and error bars are fold-change means ± s.d. of two biological replicates.

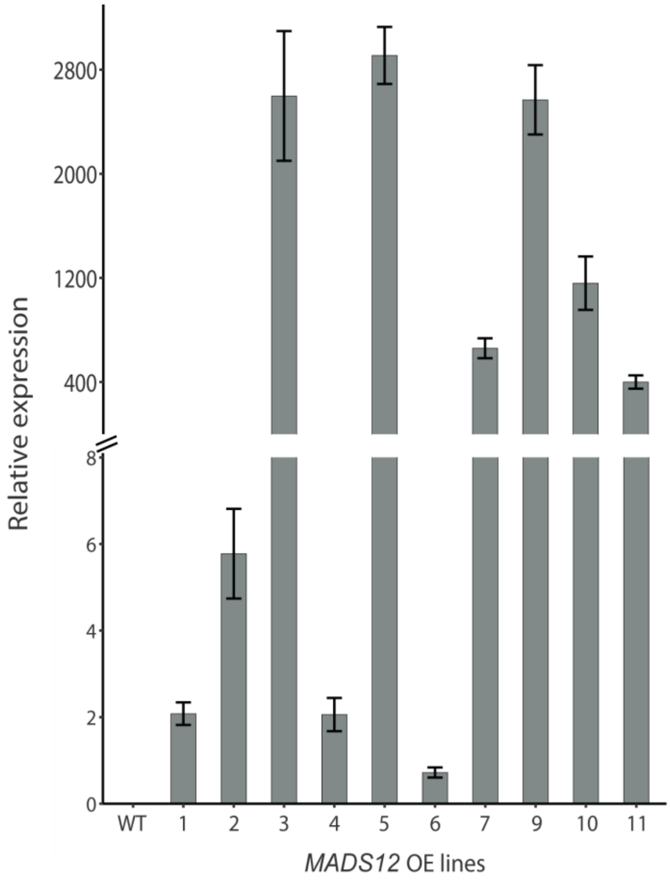

Figure S3. Phenological studies of *MADS12* OE3 and OE5 overexpressing plants. (A, B) Growth cessation scoring (A) and bud set scoring (B) of *MADS12* overexpressing OE3 and OE5 lines and WT, exposed to SD 22ºC for 8 weeks. (C) Bud burst scoring of *MADS12* overexpressing OE3 and OE5 lines and WT in response to SD 22ºC for 8 weeks and then 4 weeks of SD 4ºC. Note: 3 out of 8 *MADS12* OE5 plants reached score 1, but the rest of the plants remained at score 0. (D) Bud burst scoring of *MADS12* overexpressing OE3 and OE5 lines and WT in response to 10 weeks of SD 22ºC. Note: 3 out of 6 *MADS12* OE5 plants restored shoot growth after 25 days of LD at 22 °C. The rest of the plants remained at score 0. Values represent the mean of the measures of n= 6-15 plants. Significant differences between OEs and WT were analysed using Tukey test, * $p < 0.05$. Top panels indicate photoperiodic and temperature conditions used.

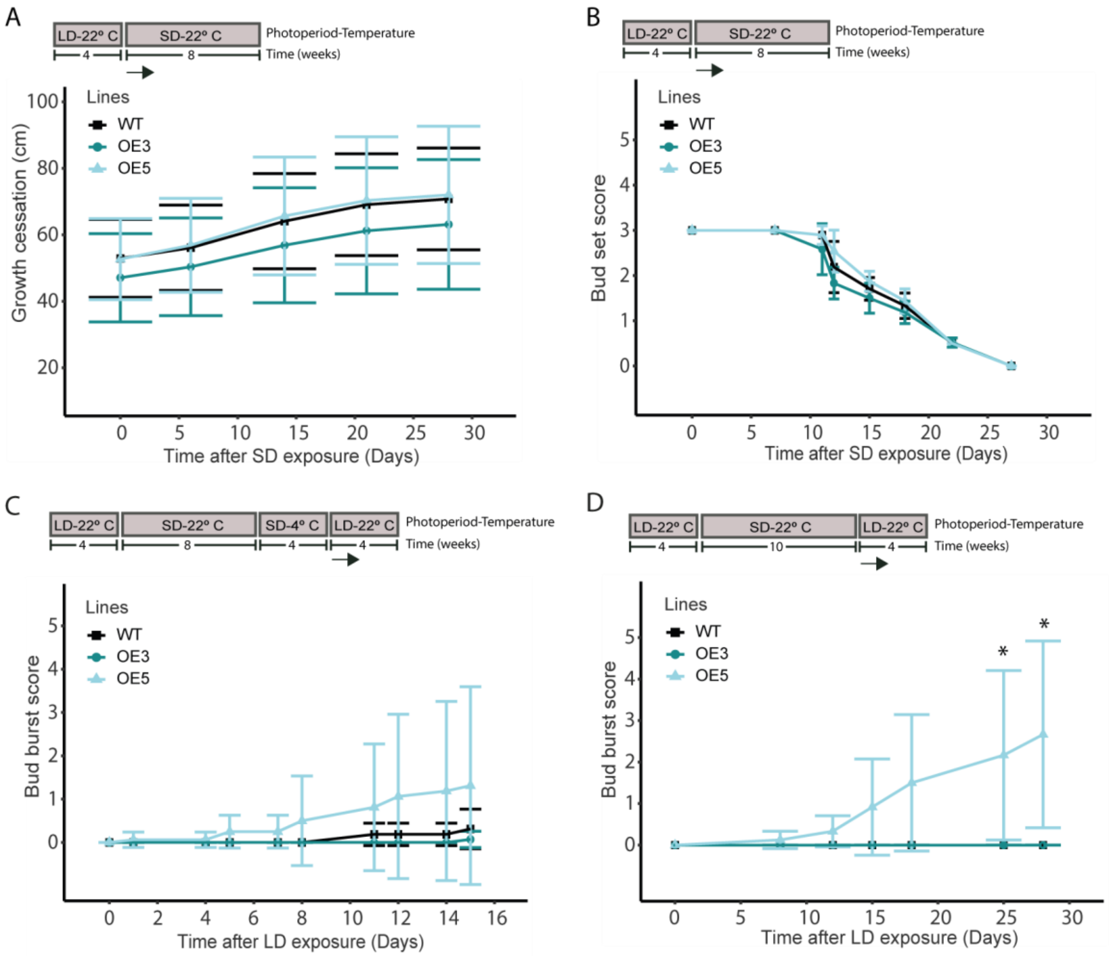

Figure S4. **Phenological studies of independent *MADS12* overexpressing plants**. Bud burst was monitored in *MADS12* overexpressing OE1, OE5, and OE7 lines and WT, exposed to SD 22ºC for 8 weeks and transferred to LD at 22°C during 25 days. Values represent the mean of the measures of n=6 plants. Top panels indicate photoperiodic and temperature conditions used.

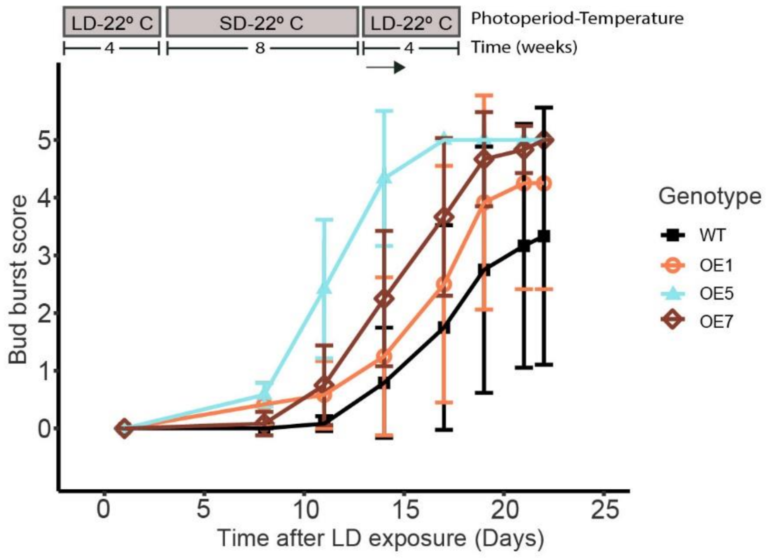

Figure S5. Heatmap showing *MADS12* coexpressed genes in apical buds during mid-winter to mid-spring period. Pearson correlation coefficient ≥0.90 was used to perform the analysis.

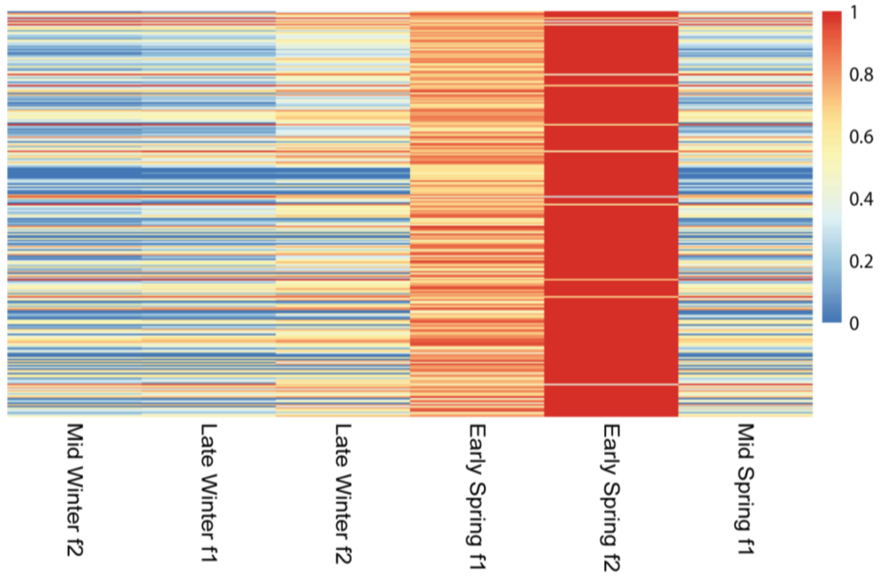

Figure S6. MADS-box *AGL16-Like* is coexpressed with *MADS12.* (A) Heatmap showing poplar *AGL16-Like* and *MADS12* pattern of gene expression in apical buds during mid-winter to mid-spring period. (B) qRT-PCR analysis of poplar *AGL16-Like gene*, in ecodormant *MADS12* overexpressing OE3 and OE5 lines and WT apices collected after 5 days in LD 22 °C treatment. *Ubiquitin7* is used as the housekeeping gene. Plotted values and error bars are fold-change means ± s.d. of two biological replicates. Asterisks (*) represent statistical differences assessed by one way ANOVA followed by Tukey post hoc test (p <0.05).

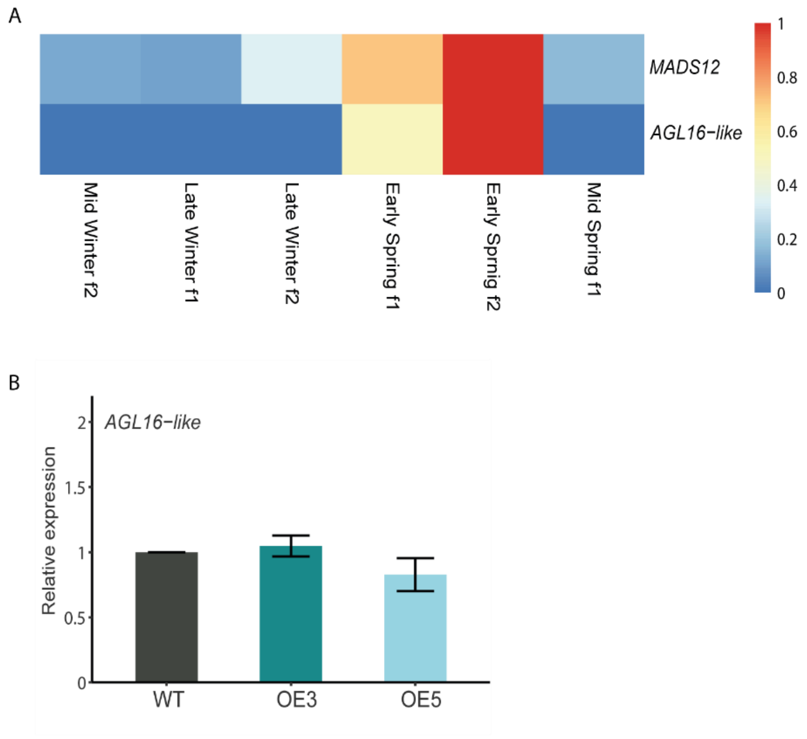

Figure S7. ***PIN5b* is downregulated in *MADS12* overexpressing lines during ecodormancy**. (A) qRT-PCR analysis of *PIN5b*, in ecodormant *MADS12* overexpressing OE3 and OE5 lines and WT apices collected after 5 days in LD 22 °C treatment. *Ubiquitin7* is used as the housekeeping gene. Plotted values and error bars are fold-change means ± s.d. of two biological replicates. Asterisks (*) represent statistical differences assessed by one way ANOVA followed by Tukey post hoc test (p <0.05).

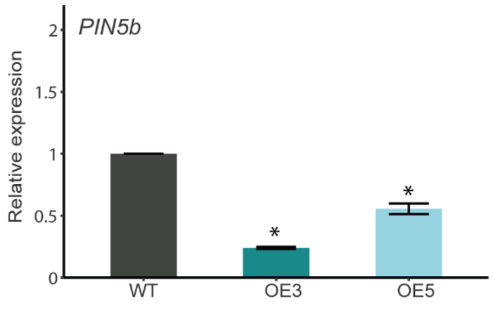

Figure S8. Heatmap showing the expression pattern during mid-winter to mid-spring period of hybrid poplar *SOC1 like* genes, including *MADS12*, *GA20ox3, GA20ox5, GA20ox6* and *SVL* in apical buds.

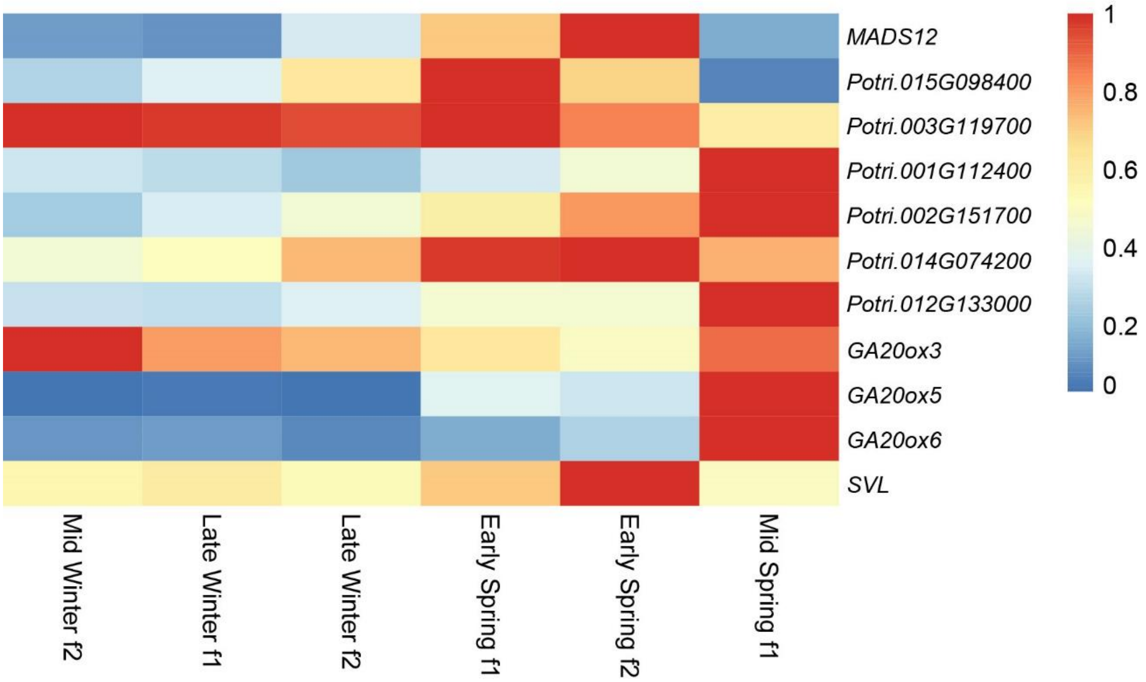

Table S1. List of primers used.

Gene	Gene ID	Primer name	Sequence
MADS12	Potri.012G100200	P012_GW	GGGGACAAGTTTGTACAAAAAAGCAGGCTTCATGGGGAGAGGGAAAGTGGAGC
MADS12	Potri.012G100200	P012_GW	GGGGACCACTTTGTACAAGAAAGCTGGGTCCTCTTTAATGGTGAGATCCATCATC
MADS12	Potri.012G100200	P012_qPCR_fwd	AGCACGTCAACTGGCTGAAAG
MADS12	Potri.012G100200	P012_qPCR_rev	ATCCATCATCAATCGTCCTCAG
CYC6	(Karlberg *et al.*, 2011)	CYC6_qPCR_fwd	AAGGGTTTCTTGCAACTTCG
CYC6	(Karlberg *et al.*, 2011)	CYC6_qPCR_rev	CATGGCTTTGGTTGAGGAAT
Ubiquitin7	Potri.005G198700	UBQ7_fwd	GGAACGGGTTGAGGAGAAAGAAG
Ubiquitin7	Potri.005G198700	UBQ7_rev	GCAAGAACAAGATGAAGCACAGAGC
FT1	(Hsu *et al.*, 2012)	FT1_qPCR_fwd	CAACTGGGGCAAGCTTTGGCCATGAAAC
FT1	(Hsu *et al.*, 2012)	FT1_qPCR_rev	TTATCGCCTCCTACCACCAGAGCCAC
GA2ox3	Potri.004G065000	GA2ox3_qPCR_fwd	GGACCTCCTAACCCTTTTGG
GA2ox3	Potri.004G065000	GA2ox3_qPCR_rev	CAGCAGAGCGGAAAATCTGTGG
GA2ox4	Potri.008G101600	GA2ox4_qPRC_fwd	AGGTAGGGTTTGGAGAGCAT

GA2ox4	Potri.008G101600	GA2ox4_qPCR_rev	GGTAGCGGGATCAGGTGTTA
GA2ox5	Potri.010G149700	GA2ox5_qPCR_fwd	GCACCCCCACTTAATGCAAG
GA2ox5	Potri.010G149700	GA2ox5_qPCR_rev	TATCTCCAAGTCGCAGAGCA
PIN5	(S. Zheng *et al.*, 2020)	PIN5_qPCR_fwd	TCCTTTCCAAGGTGCTCACT
PIN5	(S. Zheng *et al.*, 2020)	PIN5_qPCR_rev	CACTAATGCAACGTAGAGTGGT
AGL16	Potri.002G109700	AGL16_qPCR_fwd	AAAGCGAAAGAGCTGGCGAT
AGL16	Potri.002G109700	AGL16_qPCR_rev	CTGACCTGGAGCTGGAGAAA

Table S2. Hybrid poplar *GA2ox4* promoter analysis. Identification of MADS-box TF binding sites using the Plant Pan 3.0 resource (http://plantpan.itps.ncku.edu.tw). TF ID indicates the TF that putative bind to the query promoter sequence.

Position	Strand	Binding sequence	TF ID
1612	+	ttttaaaaagGGAAA	POPTR_0001s08510;POPTR_0001s13660; POPTR_0001s29100;POPTR_0001s33600; POPTR_0002s10570;POPTR_0002s15310; POPTR_0003s16800;POPTR_0003s16840; POPTR_0003s16870;POPTR_0004s06310; POPTR_0004s11430;POPTR_0004s11450; POPTR_0006s04730;POPTR_0007s03270; POPTR_0007s03290;POPTR_0007s14300; POPTR_0008s09780;POPTR_0009s06060; POPTR_0010s16390;POPTR_0011s03150; POPTR_0012s05950;POPTR_0012s10190; POPTR_0013s09960;POPTR_0013s10190; POPTR_0014s07020;POPTR_0015s11030; POPTR_0015s14010;POPTR_0017s13400
1330	-	gtgctatTTTTGgta	POPTR_0002s02990;POPTR_0005s12000; POPTR_0007s13660;POPTR_0013s09960
327	+	cacgctcataaaaAGAAAttt	POPTR_0001s08510;POPTR_0001s13660; POPTR_0002s15310;POPTR_0003s16820; POPTR_0003s16840;POPTR_0003s16850; POPTR_0003s16870;POPTR_0004s11450; POPTR_0006s04730;POPTR_0008s09780; POPTR_0014s07020
1562	+	ggtggtaataaatAGAAAgtg	POPTR_0001s08510;POPTR_0001s13660; POPTR_0002s15310;POPTR_0003s16820; POPTR_0003s16840;POPTR_0003s16850; POPTR_0003s16870;POPTR_0004s11450; POPTR_0006s04730;POPTR_0008s09780; POPTR_0014s07020

1614	+	ttaaaaagGGAAAt	POPTR_0002s07920;POPTR_0005s20480
76	+	aCAAAAatgaa	POPTR_0001s08510;POPTR_0001s13660;<br>POPTR_0001s33600;POPTR_0002s09290;<br>POPTR_0002s10570;POPTR_0002s15310;<br>POPTR_0003s16800;POPTR_0003s16820;<br>POPTR_0003s16840;POPTR_0003s16850;<br>POPTR_0003s16870;POPTR_0004s06310;<br>POPTR_0004s11430;POPTR_0004s11450;<br>POPTR_0006s04730;POPTR_0007s14300;<br>POPTR_0008s09780;POPTR_0009s06060;<br>POPTR_0010s16390;POPTR_0011s03150;<br>POPTR_0012s05950;POPTR_0012s10190;<br>POPTR_0012s14770;POPTR_0013s09960;<br>POPTR_0013s10190;POPTR_0014s07020;<br>POPTR_0015s11030;POPTR_0015s14010;<br>POPTR_0017s13400
455	-	ttcatTTTTGt	POPTR_0001s08510;POPTR_0001s13660;<br>POPTR_0001s33600;POPTR_0002s09290;<br>POPTR_0002s10570;POPTR_0002s15310;<br>POPTR_0003s16800;POPTR_0003s16820;<br>POPTR_0003s16840;POPTR_0003s16850;<br>POPTR_0003s16870;POPTR_0004s06310;<br>POPTR_0004s11430;POPTR_0004s11450;<br>POPTR_0006s04730;POPTR_0007s14300;<br>POPTR_0008s09780;POPTR_0009s06060;<br>POPTR_0010s16390;POPTR_0011s03150;<br>POPTR_0012s05950;POPTR_0012s10190;<br>POPTR_0012s14770;POPTR_0013s09960;<br>POPTR_0013s10190;POPTR_0014s07020;<br>POPTR_0015s11030;POPTR_0015s14010;<br>POPTR_0017s13400
811	-	tatttTTTTGg	POPTR_0001s08510;POPTR_0001s13660;<br>POPTR_0001s33600;POPTR_0002s09290;<br>POPTR_0002s10570;POPTR_0002s15310;<br>POPTR_0003s16800;POPTR_0003s16820;<br>POPTR_0003s16840;POPTR_0003s16850;<br>POPTR_0003s16870;POPTR_0004s06310;<br>POPTR_0004s11430;POPTR_0004s11450;<br>POPTR_0006s04730;POPTR_0007s14300;<br>POPTR_0008s09780;POPTR_0009s06060;<br>POPTR_0010s16390;POPTR_0011s03150;<br>POPTR_0012s05950;POPTR_0012s10190;<br>POPTR_0012s14770;POPTR_0013s09960;<br>POPTR_0013s10190;POPTR_0014s07020;<br>POPTR_0015s11030;POPTR_0015s14010;<br>POPTR_0017s13400
1332	-	gctatTTTTGg	POPTR_0001s08510;POPTR_0001s13660;<br>POPTR_0001s33600;POPTR_0002s09290;<br>POPTR_0002s10570;POPTR_0002s15310;<br>POPTR_0003s16800;POPTR_0003s16820;<br>POPTR_0003s16840;POPTR_0003s16850;

			POPTR_0003s16870;POPTR_0004s06310; POPTR_0004s11430;POPTR_0004s11450; POPTR_0006s04730;POPTR_0007s14300; POPTR_0008s09780;POPTR_0009s06060; POPTR_0010s16390;POPTR_0011s03150; POPTR_0012s05950;POPTR_0012s10190; POPTR_0012s14770;POPTR_0013s09960; POPTR_0013s10190;POPTR_0014s07020; POPTR_0015s11030;POPTR_0015s14010; POPTR_0017s13400
1729	+	cCAAAAaagca	POPTR_0001s08510;POPTR_0001s13660; POPTR_0001s33600;POPTR_0002s09290; POPTR_0002s10570;POPTR_0002s15310; POPTR_0003s16800;POPTR_0003s16820; POPTR_0003s16840;POPTR_0003s16850; POPTR_0003s16870;POPTR_0004s06310; POPTR_0004s11430;POPTR_0004s11450; POPTR_0006s04730;POPTR_0007s14300; POPTR_0008s09780;POPTR_0009s06060; POPTR_0010s16390;POPTR_0011s03150; POPTR_0012s05950;POPTR_0012s10190; POPTR_0012s14770;POPTR_0013s09960; POPTR_0013s10190;POPTR_0014s07020; POPTR_0015s11030;POPTR_0015s14010; POPTR_0017s13400
